# Humeral Metadiaphyseal Fracture With Severe Osteoarthritis of the Shoulder and Simultaneous Contralateral Proximal Humeral Fracture Treated With Bilateral Reverse Shoulder Arthroplasty: A Rare Case Report

**DOI:** 10.7759/cureus.73166

**Published:** 2024-11-06

**Authors:** Gaku Matsuzawa, Taku Hatta, Toshitake Aizawa

**Affiliations:** 1 Department of Orthopaedic Surgery, Iwaki City Medical Center, Iwaki, JPN; 2 Department of Orthopaedic Surgery, Tome Citizen Hospital, Tome, JPN; 3 Department of Orthopaedic Surgery, Joint Surgery, Sports Clinic Ishinomaki, Ishinomaki, JPN

**Keywords:** bilateral, humerus fracture, metadiaphysis, osteoarthritis, proximal, reverse shoulder arthroplasty

## Abstract

Simultaneous bilateral proximal humerus fractures (PHFs) are infrequent. Particularly rare are cases of PHFs extending to the metadiaphysis accompanied by severe osteoarthritis (OA) of the shoulder. To our knowledge, there have been no reports of bilateral fractures that include metadiaphyseal fractures with severe OA on one side and a PHF on the contralateral side, treated with bilateral reverse shoulder arthroplasty (RSA). We present a case of an 84-year-old woman with a right humeral metadiaphyseal fracture with severe OA and a left proximal humeral fracture, both treated with bilateral RSA. The surgical technique for the humerus metadiaphyseal fracture involved adjusting soft-tissue tension for RSA, where the proximal bone fragment was opened and wrapped around the humeral component. The clinical outcomes of this case suggest that this surgical technique can be a viable option to restore function in rare cases of humeral metadiaphyseal fractures with severe OA of the shoulder and simultaneous contralateral proximal humeral fractures.

## Introduction

Proximal humerus fractures (PHFs) are more likely to occur due to falls in older adults with poor bone quality and are the fourth most common fracture caused by falls in this population [1]. It is known that simultaneous bilateral PHFs are relatively infrequent, and to date, there have been several case reports involving a small number of patients [2,3]. Treating older patients with PHFs using open reduction and internal fixation (ORIF) can be difficult because of their poor bone quality. Particularly, achieving rigid reduction of the proximal humerus fragments around the humeral head is challenging [4]. Additionally, a clinical study indicated that one-third of PHF cases may be accompanied by shoulder osteoarthritis (OA) [5]. Among surgical options involving shoulder prostheses, hemiarthroplasty (HA) remains difficult because the postoperative outcome of this procedure can be significantly affected by the position of the insufficiently reduced tuberosity fragments [6]. On the other hand, reverse shoulder arthroplasty (RSA) has the advantage of using the deltoid muscle to achieve shoulder elevation [7], and better clinical outcomes have been reported for RSA compared to those of ORIF and HA [8]. Therefore, RSA is expected to be an effective treatment, especially for older patients with comminuted PHFs.

There are few reports on treatment using ORIF [9] or implants [10] for cases of PHFs extending to the metadiaphysis; however, careful implant selection is required [11]. Cases of PHF extending to the metadiaphysis with severe OA of the shoulder are extremely rare, and few reports are available as references for the treatment of this fracture type. Although simultaneous PHFs can occur in patients with seizures [12] or poor bone quality [3,13], reports on these injuries are scarce. Furthermore, there are no reports of humeral metadiaphyseal fractures with shoulder OA or simultaneous contralateral PHF treated with bilateral RSA as far as we are aware. For older patients, besides bone fusion at the fracture site, early recovery of upper limb function is crucial for improvement in activities of daily living (ADLs), such as using a cane for walking.

The purpose of this case report is to present the outcomes of simultaneous right metadiaphyseal and left proximal humeral fractures treated with RSA in an older woman. The patient gave her informed consent to the surgical procedure as well as to the publication of this report.

## Case presentation

**Figure 1:**
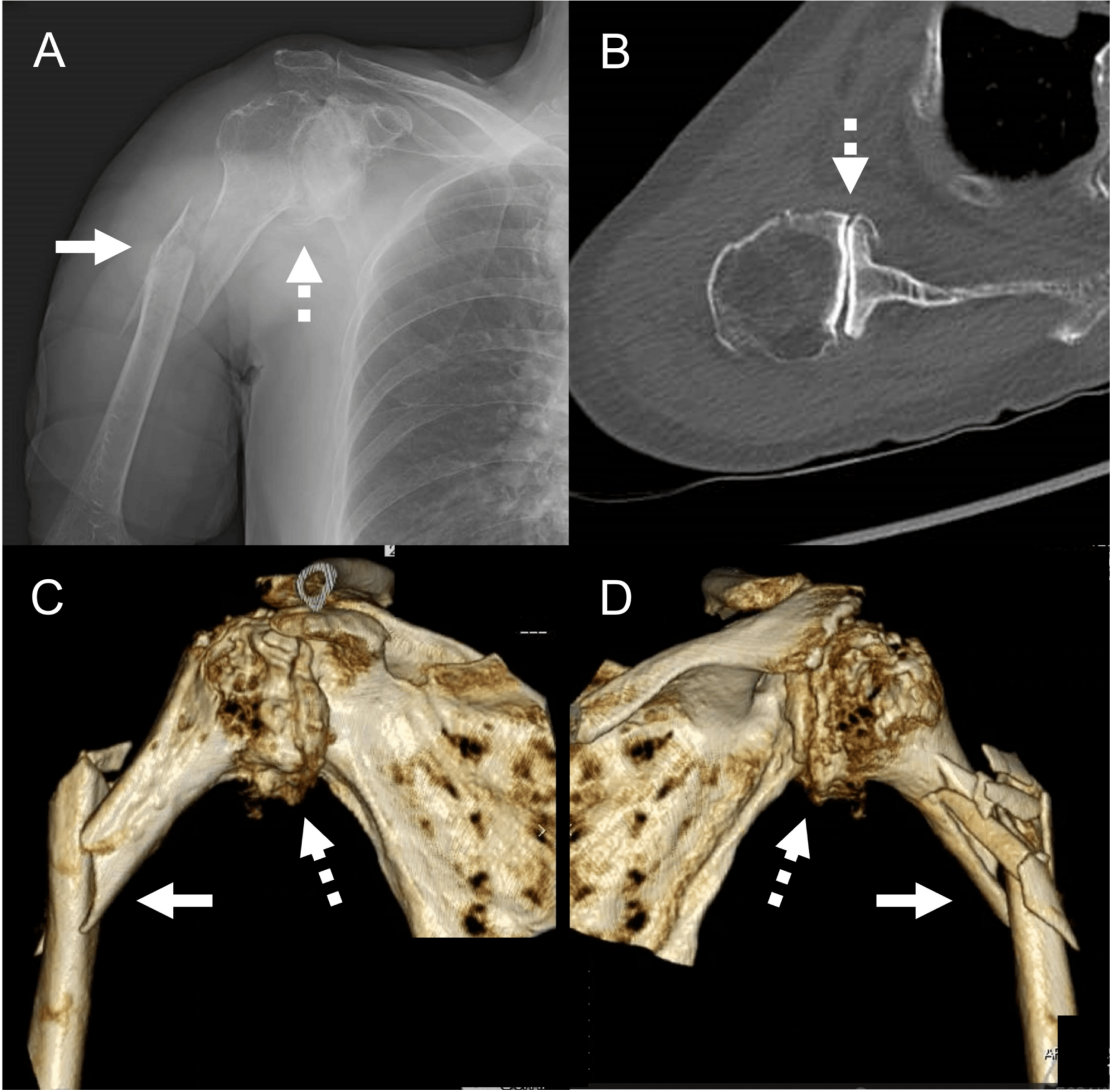
The preoperative images of the right shoulder. The preoperative images of the right shoulder include anteroposterior X-ray (A), axial view of CT (B), anterior view of 3D-CT (C), and posterior view of 3D-CT (D). These images show a metadiaphyseal humeral fracture (indicated by an arrow) with severe osteoarthritis (OA) (indicated by a dotted arrow).

**Figure 2:**
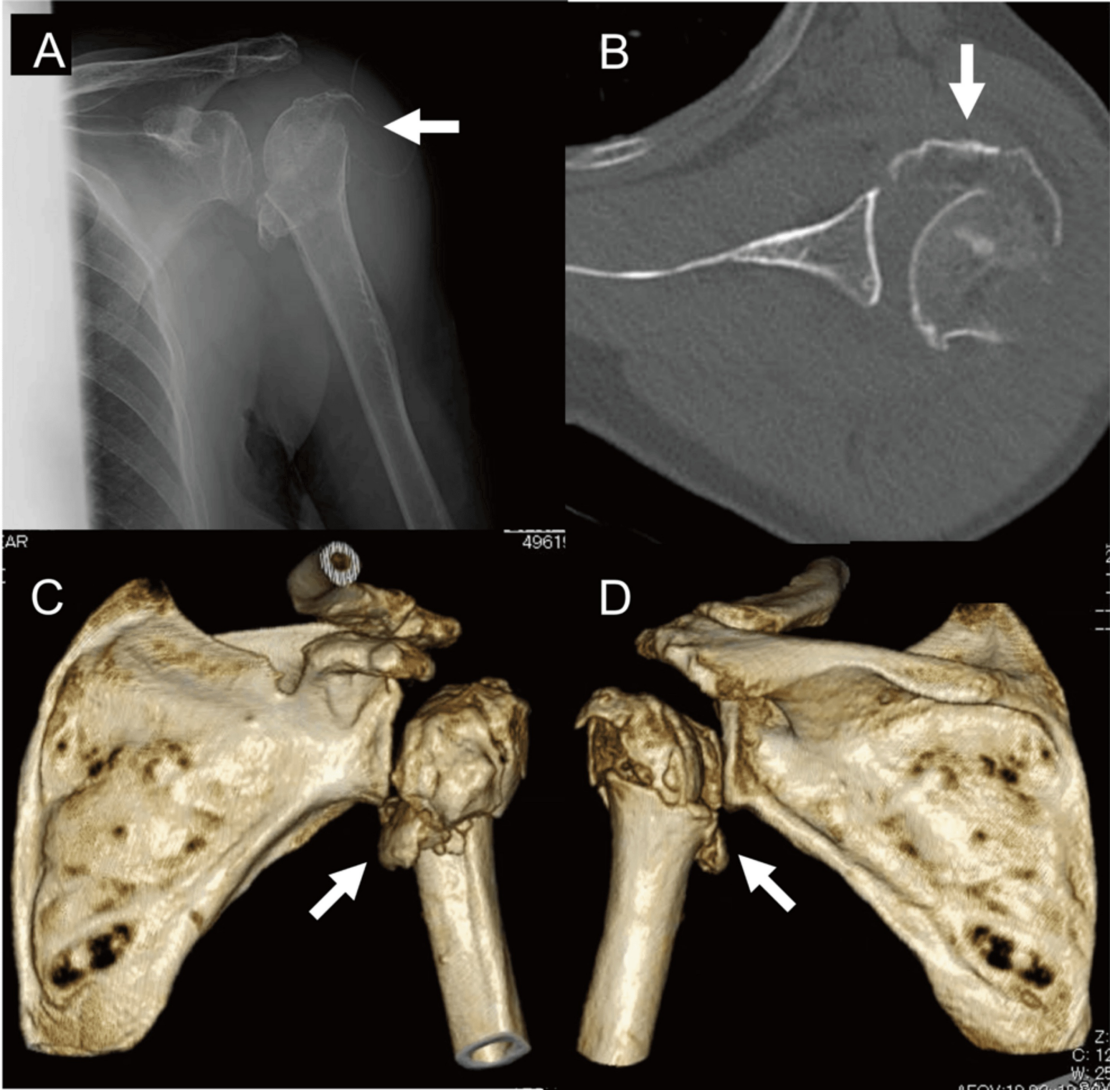
The preoperative images of the left shoulder. The preoperative images of the left shoulder include an anteroposterior X-ray (A), an axial view of CT (B), an anterior view of 3D-CT (C), and a posterior view of 3D-CT (D). These images show a proximal humeral fracture (indicated by an arrow) with osteoarthritis (OA).

An 84-year-old woman (height, 151.1 cm; weight, 52.3 kg; independent in ADL; right-hand-dominant), whose hobbies included ballroom dancing, had chronic bilateral shoulder pain for >30 years and difficulty elevating her right shoulder for >5 years. She claimed to have fallen while gardening, initially hitting her right shoulder, before falling onto her left-hand side due to recoil. The patient had a history of hypertension and subdural hematoma. She had no history of smoking or alcohol consumption. The patient was admitted to the emergency room. She complained of pain in both upper limbs. Swelling was observed in both arms. Neurovascular deficits were not observed. The patient underwent a whole-body trauma check-up in the ER, and other injuries were ruled out. Radiographs showed a right metadiaphysial humeral fracture (AO Foundation/Orthopaedic Trauma Association classification: AO/OTA 12C3) with OA of Hamada grade 4a (Figures 1A-1D) and a left Neer 4-part PHF (AO/OTA 11C3.1) with an OA of Hamada grade 2 (Figure 2A-2D). Right RSA (Aequalis^Ⓡ^ Reversed FX; Stryker, Inc., Kalamazoo, MI) was performed 12 days after the injury, followed by left RSA (Aequalis^Ⓡ^ Reversed FX; Stryker, Inc., Kalamazoo, MI) one week later.

**Figure 3:**
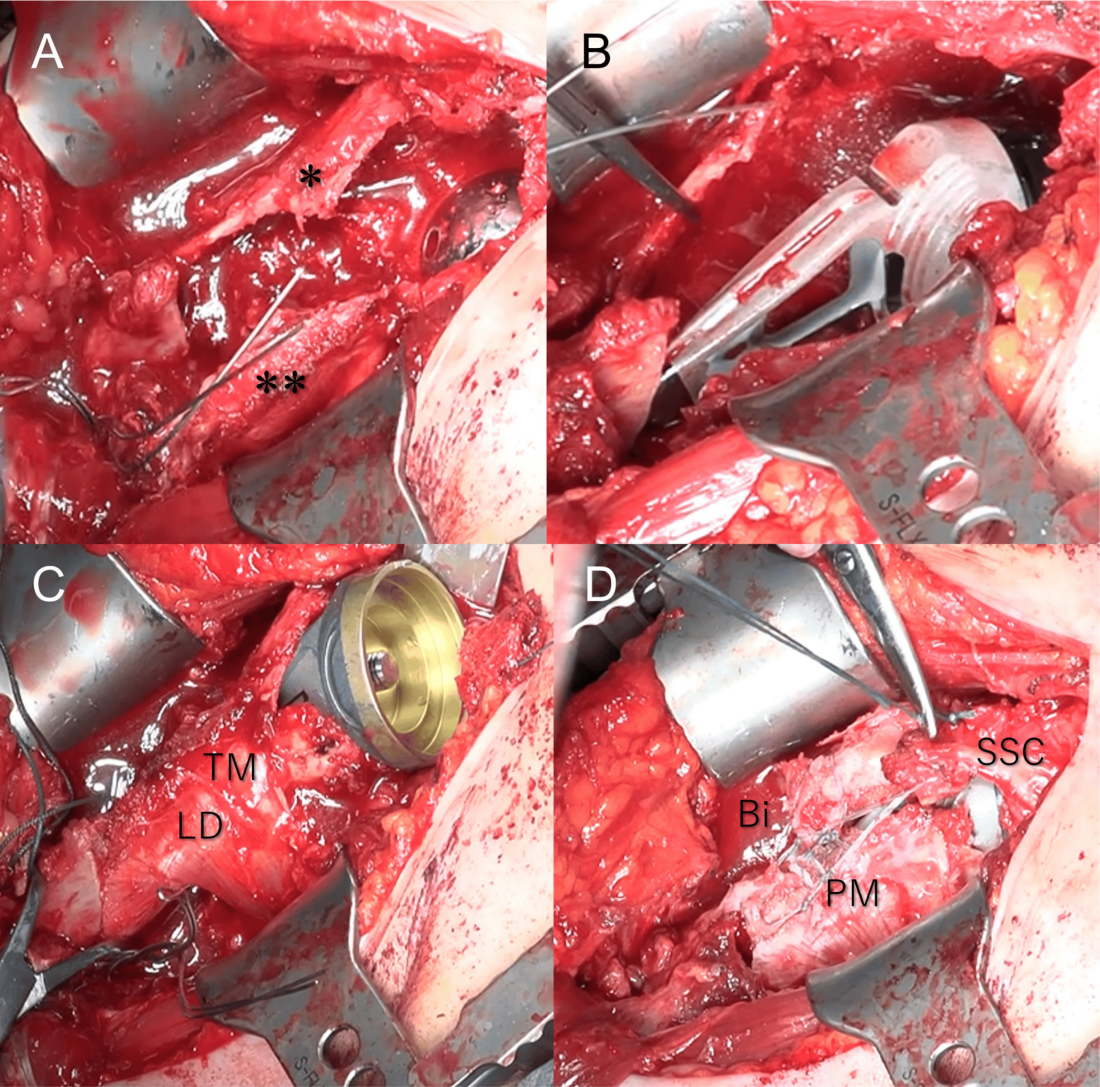
The operative procedure of the right shoulder. The proximal bone fragment was opened at the biceps groove level (A) and divided into the medial and lateral fragments. The humeral implant was placed in the distal fragment (B). The proximal fragments were wrapped around the component (C). The resected muscles were repaired at nearly their original sites (D). *: Lateral part of the proximal fragment, **: Medial part of the proximal fragment, TM: Teres major, LD: Latissimus dorsi, SSC: Subscapularis, Bi: Biceps, PM: Pectoralis major.

Right shoulder surgery was performed using a deltopectoral approach. The long head of the biceps (LHBT) was cut at the level of the bicep groove; the distal part was turned on, and the proximal part was resected. The supraspinatus (SSP) was defected. The proximal part of the pectoralis major (PM) was detached at a level where the bone fragments could be identified. Dislocating the shoulder was difficult due to severe degeneration; therefore, the lesser tuberosity (LT) was split with the subscapularis (SSC) attached, and the flattened and degenerated humeral head was resected at the level of the anatomical neck to manipulate the humerus. Through this procedure, the proximal fragment became a mass with the infraspinatus (ISP) and teres minor (Tm) attached to the greater tuberosity (GT), latissimus dorsi (LD), and teres major (TM). The glenoid baseplate and glenosphere were placed first, with the position of the inferior margin of the baseplate at the same height as that of the glenoid. The humeral implant was placed through the medullary canal from the proximal to the distal bone fragment using the so-called “shish-kebab” technique, but reduction was not possible due to excessive tension. Thus, the proximal bone fragment was opened at the biceps groove level and divided into the medial fragment with the LD, TM, and PM and the lateral fragment with the ISP and Tm instead of shortening the humerus (Figure 3A). The deltoid was attached to the distal bone fragments. The humeral implant was positioned in the distal fragment with cement at 20° retroversion to achieve the ideal tension for repositioning (Figure 3B). Proximal fragments were wrapped around the component firmly with an autologous bone graft using strong sutures, and the detached muscles such as the LHBT, PM, and SSC, were ideally repaired (Figures 3C-3D).

Left shoulder surgery was also performed using the deltopectoral approach. LT with SSC was secured, as well as GT with SSP and ISP. Both glenoid and humeral components were placed, and LT and GT were ideally repaired.

**Figure 4:**
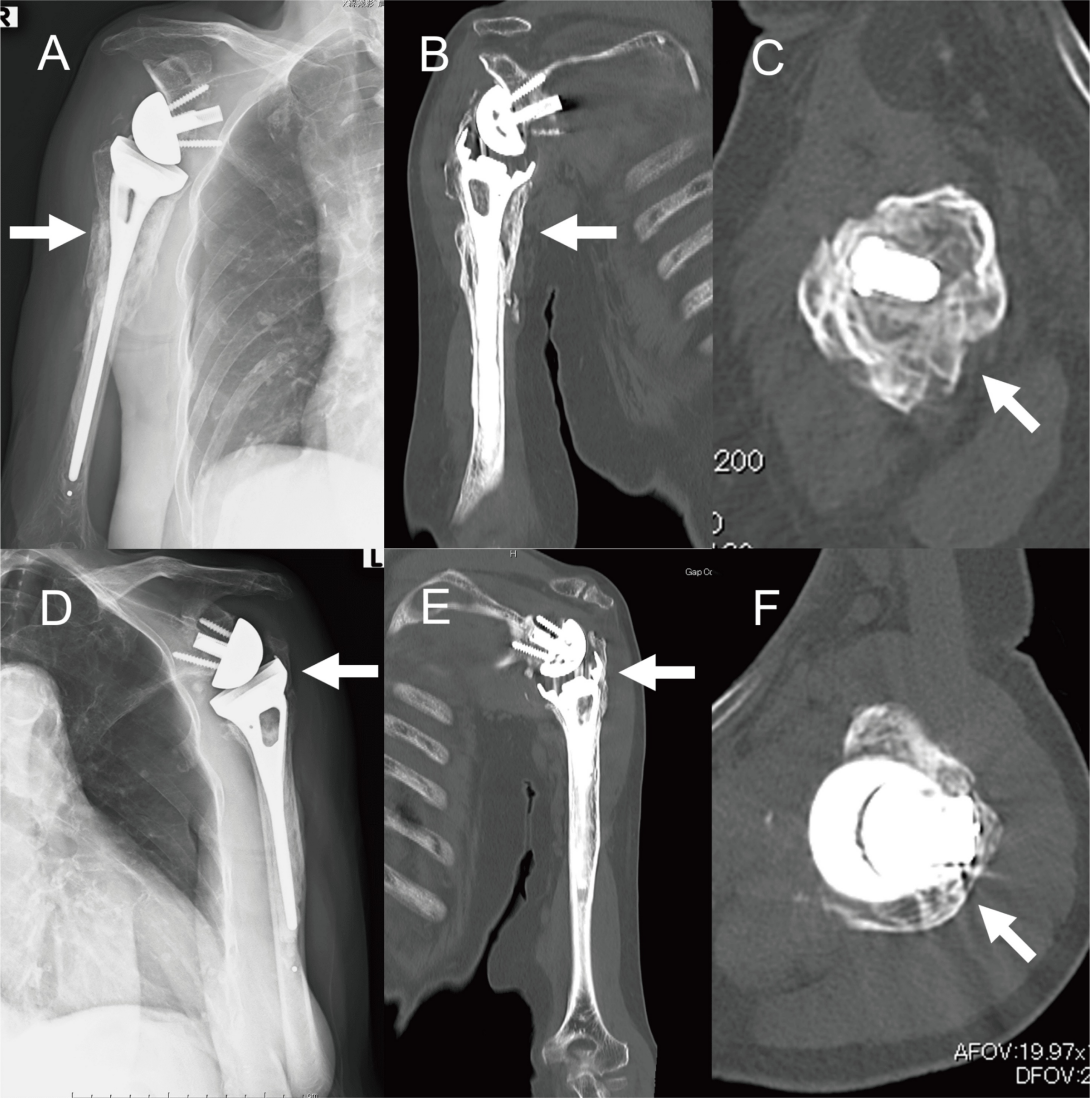
The final follow-up images. The shoulder images of left anteroposterior X-ray (A), coronal view of CT (B), axial view of CT (C), right anteroposterior X-ray (D), coronal view of CT (E), and axial view of CT (F). The fragments of both shoulders were ideally healed (indicated by arrows).

**Figure 5:**
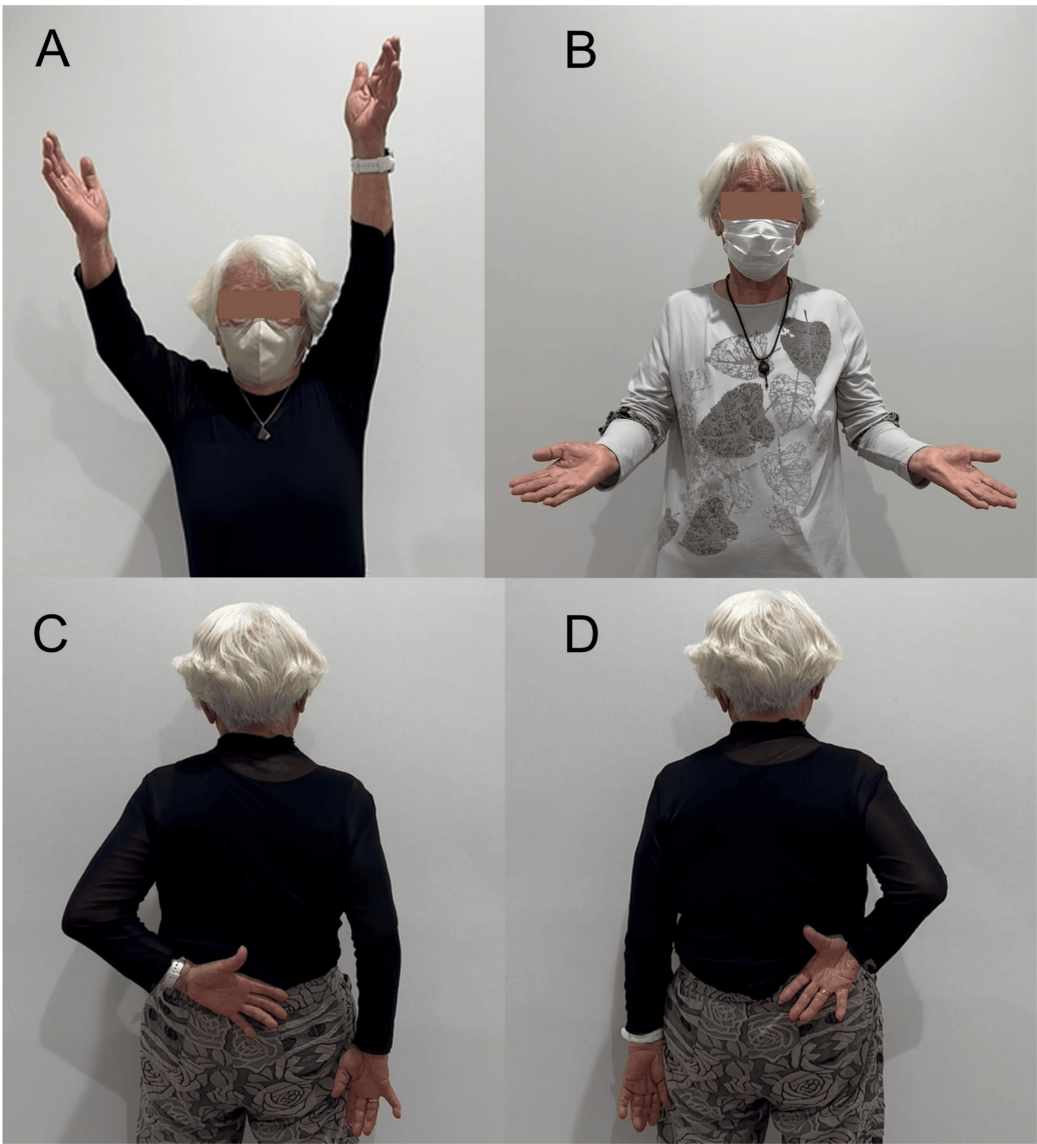
The final follow-up of shoulder function. Postoperative function of both shoulders, including active elevation (A), external rotation (B), and internal rotation for the left (C) and right (D) shoulders, was satisfactory.

The arms were immobilized for four weeks postoperatively with a shoulder immobilizer, except during rehabilitation. Both shoulders underwent the same rehabilitation program. Pendulum, passive range of motion (ROM) exercises in the scapular plane, and isometric deltoid muscle exercises were started one week postoperatively. Active ROM exercises, including external and internal rotation, were started after sling immobilization. At the final follow-up (four years postoperatively), the fragments in both shoulders had healed (Figures 4A-4D). The patient’s active ROM was as follows: right and left forward elevations, 140° and 160°, respectively; external rotations with the arm at the side, 30° and 40°, respectively; and internal rotations, L1 and L3, respectively (Figures 5A-5D). The right and left American Shoulder and Elbow Surgeons scores were 90 and 92 points, respectively, and the Constant scores were 85 and 87 points, respectively. There was no damage to any nerve on either side in this case. She enjoyed ballroom dancing for fun.

## Discussion

Because PHF in older women is rooted in bone fragility, it may be difficult to fuse tubercle bone fragments, especially in cases of comminuted PHF, and postoperative treatment outcomes for ORIF and HA vary greatly across cases [5,7]. However, RSA can elevate the function of the shoulder joint without relying on the quality of the rotator cuff; therefore, it is effective for treating proximal humeral fractures with great tension for the fusion of large and small tubercle bone fragments [14]. In this report, both humeral fractures in this older woman were treated with RSA to start rehabilitation early and achieve stable shoulder joint function after surgery. Good treatment outcomes were observed in this case. On the right side, in the case of a metadiaphyseal fracture with OA, there was a problem with ideal RSA tension adjustment in addition to the length and fixation method needed to stabilize the implant. In cases of severe OA of the shoulder, the position of the humeral head is relatively proximate and medialized compared to the normal shoulder for a long period, such that the total distance of distalization and lateralization of the humerus in the conventional RSA settings might be relatively longer than in normal cases, and this would cause a larger difference in the length of the deltoid and rotator cuff muscles before and after surgery. Excessive muscle tension caused by over-distalization and lateralization in RSA can be avoided because of the risk of acromial fracture [15] and humeral postoperative stress fracture [16].

In this case, we needed to take sufficient care with the humeral implant fixation because we could use only the long implant that must be fixed distally with cement, or we could not use a long cementless humeral component at the time of surgery. In this case, stabilizing the humeral component required firm fixing to the distal bone fragment because the proximal bone fragment was free. Using the method of penetrating the proximal bone fragment like a “shish-kebab” [17], it is difficult to inject cement into the distal bone fragment and bypass the proximal fragment, especially when the proximal bone fragment is long, and extracting the cement exposed outside from bone is difficult. Tension in the RSA may be adjusted by shortening the humerus. Humeral shortening includes a cut of the humeral fragments only in the proximal part, proximal and distal parts, only the distal part, or overlapping of the bone fragment around the implant after the implant is placed. Osteotomy may be difficult in some cases because it must be performed in a position that preserves the attachment of the external rotator muscles and other muscles that are important for upper-extremity function. Osteotomy is time-consuming if it must be adjusted several times until proper tension is achieved, so we selected an alternative method to it. By splitting the proximal bone fragment longitudinally and placing the implant and distal bone segment in a position that achieves appropriate tension in the elongation direction, we shortened the humerus instead of performing osteotomy. If the bone fragments are placed as an “open book,” like in this case, it is necessary to obtain bony fusion after the reduction of the fragments. In this case, the implant was firmly fixed to the distal humeral fragment using bone cement with the appropriate tension of the RSA, and then the “open book” bone fragments were wrapped around the implant, putting cancellous bone between the fragments and implant. The cancellous bone was taken from the excised humeral head in cases of fracture. Good bone fusion and appropriate RSA tension were achieved by using this procedure. Regarding the right shoulder of our patient, one therapeutic approach involves performing ORIF followed by RSA once bone fusion has been achieved to mitigate the difficulties associated with surgery. However, this strategy is associated with a potential decrease in postoperative clinical outcomes and an increase in the incidence of complications, including infection, compared with initial RSA [18]. Thus, if possible, the performance of a single surgical procedure capable of achieving a satisfactory treatment result is preferred in order to ensure patient safety.

One issue of concern with simultaneous bilateral RSA implantation is the complete temporary disability of the patient and the conceivable complexity of performing proper physical therapy [19]. However, good clinical outcomes have been reported for both simultaneous bilateral and staged bilateral total shoulder arthroplasty [20] due to the shortening of resting periods. Regarding surgical invasiveness, simultaneous bilateral RSA may result in a larger amount of bleeding and longer anesthesia time. On the other hand, if staged bilateral RSA is performed with a one-week interval, the disadvantages include having to undergo anesthesia twice and a longer overall duration of treatment. However, advantages such as reduced blood loss and shorter anesthesia time per surgery are also noted. Since both methods have their benefits, the choice of which method to use should be based on the patient’s background and objectives. We performed staged RSA at one-week intervals for a bilateral humerus fracture case, and the clinical outcomes were good for both shoulders, as previously reported. However, it should be noted that the procedure described herein was performed under very limited conditions, namely in an elderly individual with the presence of OA and bilateral onset. Our findings suggest that the proposed approach is a good option for the type of fracture reported in this study.

## Conclusions

This case report describes a treatment option involving short-interval staged RSA for a rare injury that includes a simultaneous metadiaphyseal humerus fracture and a proximal four-part humerus fracture. We outline the treatment strategies employed. The clinical outcomes of this case indicate that this surgical technique is a viable option to restore function in rare cases of humeral metadiaphyseal fractures with severe OA of the shoulder and simultaneous contralateral proximal humeral fractures.

## References

[1] Court-Brown CM, Clement ND, Duckworth AD, Biant LC, McQueen MM (2017). The changing epidemiology of fall-related fractures in adults. Injury.

[2] Tokuhiro T, Urita A, Kameda Y, Motomiya M, Watanabe N, Iwasaki N (2022). Simultaneous bilateral proximal humerus fractures treated with single-stage bilateral reverse shoulder arthroplasty. Case Rep Orthop.

[3] Almaghrabi RA, Almousa AM, Almulla A, Salem O, Almana L (2023). Single-stage bilateral reverse shoulder arthroplasty for a bilateral four-part fracture dislocation of the proximal humerus in an elderly patient: a case report. Cureus.

[4] Jost B, Spross C, Grehn H, Gerber C (2013). Locking plate fixation of fractures of the proximal humerus: analysis of complications, revision strategies and outcome. J Shoulder Elbow Surg.

[5] Klement MR, Nickel BT, Bala A, Penrose CT, Zura RD, Garrigues GE (2016). Glenohumeral arthritis as a risk factor for proximal humerus nonunion. Injury.

[6] Boileau P, Krishnan SG, Tinsi L, Walch G, Coste JS, Molé D (2002). Tuberosity malposition and migration: reasons for poor outcomes after hemiarthroplasty for displaced fractures of the proximal humerus. J Shoulder Elbow Surg.

[7] Boileau P, Watkinson DJ, Hatzidakis AM, Balg F (2005). Grammont reverse prosthesis: design, rationale, and biomechanics. J Shoulder Elbow Surg.

[8] Yahuaca BI, Simon P, Christmas KN, Patel S, Gorman RA 2nd, Mighell MA, Frankle MA (2020). Acute surgical management of proximal humerus fractures: ORIF vs. hemiarthroplasty vs. reverse shoulder arthroplasty. J Shoulder Elbow Surg.

[9] Robinson CM, Stirling PH, MacDonald DJ, Strelzow JA, Goudie EB (2020). Open reduction and long locking plate fixation of complex proximal humeral metadiaphyseal fractures. J Bone Joint Surg Am.

[10] Helal A, Heimdal T, Lo EY (2023). Arthroplasty as primary treatment for metadiaphyseal proximal humerus fractures: a viable alternative to osteosynthesis for the elderly. J Shoulder Elb Arthroplast.

[11] Greiner S, Uschok S, Herrmann S, Gwinner C, Perka C, Scheibel M (2014). The metaphyseal bone defect predicts outcome in reverse shoulder arthroplasty for proximal humerus fracture sequelae. Arch Orthop Trauma Surg.

[12] Brackstone M, Patterson SD, Kertesz A (2001). Triple "E" syndrome: bilateral locked posterior fracture dislocation of the shoulders. Neurology.

[13] Rodriguez-Corlay RE, Velutini-Becker R, Aguilar-Alcalá LD (2016). Conservative treatment for bilateral displaced proximal humerus head fracture. Cureus.

[14] Ferrel JR, Trinh TQ, Fischer RA (2015). Reverse total shoulder arthroplasty versus hemiarthroplasty for proximal humeral fractures: a systematic review. J Orthop Trauma.

[15] Glener J, Vegas A, Schodlbauer DF, Levy JC (2024). Acromion fractures after reverse shoulder arthroplasty occur in predictable clusters. J Shoulder Elbow Surg.

[16] Giles JW, Langohr GD, Johnson JA, Athwal GS (2015). Implant design variations in reverse total shoulder arthroplasty influence the required deltoid force and resultant joint load. Clin Orthop Relat Res.

[17] Boileau P, Seeto BL, Clowez G, Gauci MO, Trojani C, Walch G, Chelli M (2020). SECEC Grammont Award 2017: the prejudicial effect of greater tuberosity osteotomy or excision in reverse shoulder arthroplasty for fracture sequelae. J Shoulder Elbow Surg.

[18] Lu V, Jegatheesan V, Patel D, Domos P (2023). Outcomes of acute vs. delayed reverse shoulder arthroplasty for proximal humerus fractures in the elderly: a systematic review and meta-analysis. J Shoulder Elbow Surg.

[19] Ceri L, Mondanelli N, Sangaletti R, Bottai V, Muratori F, Giannotti S (2019). Simultaneous bilateral reverse shoulder arthroplasty for bilateral four-part fracture of the proximal humerus in an elderly patient: a case report. Trauma Case Rep.

[20] Ajibade DA, Mourad W, Medina G, Wiater JM (2022). Simultaneous bilateral shoulder arthroplasty: a case series. J Shoulder Elbow Surg.

